# Epidemic Erysipelatous Fever at Waverly

**Published:** 1865-06

**Authors:** R. E. McVey

**Affiliations:** Waverly, Ill.


### ARTICLE XX.

### EPIDEMIC ERYSIPELATOUS FEVER AT WAVERLY, ILL. READ TO THE MEETING OF THE ILLINOIS STATE MEDICAL SOCIETY, MAY, 1865.

BY R. E. McVEY, M.D., of Waverly, Ill.

MR. PRESIDENT AND GENTLEMEN:—The paper which I shall read to you, is a brief history of an epidemic of erysipelatous fever, which occurred in and about the vicinity of Waverly, which is located near the central part of the State.

The topography of the country is high, rolling prairie, with a fine loamy soil, slightly mixed with sand; and occasional streams to drain the country sufficiently; with timber on the North and West to break the great north-western winds. The water used for domestic purposes, is obtained from wells, at the bottom of which are found a continuous bed of slate and sandstone, mixed with occasional streaks of stone coal.

The country is all in a high state of cultivation, and apparently there is no more cause for epidemics there, than in any other portion of the country. But during the winter of 1864, we had an epidemic of cerebro-spinal meningitis, which proved very intractable; and through the summer we were troubled with an epidemic of scarlatina; and when cold weather made its appearance, erysipelatous fever, which has far exceeded in fatality even cholera itself.

The cause of the epidemic seems to be obscure, but is supposed to be some atmospheric admixture, which is wholly unaccountable, so far as I know. The weather was dry and cold, mercury ranging from 32° above to 10° below 0; and no rain, scarcely, has fallen since November last, 1864, until March, 1865, making, upon the whole, a winter of the most uniform temperature, dry and pleasant, I have seen for many years, not varying from one extreme to the other, as is usually the case.

I am aware that the cause is said to be the result of the decomposition of animal and vegetable matters, the poison finding its way into the lungs with atmospheric air, or some other avenue. But it is impossible to say, for the reason that cold has the property of condensing vapors and confining them to the surface of the earth, therefore it would seem impossible for epidemics to originate from such a cause during protracted cold weather, which was the case last winter, however, it might be said that the toxic agent found its way into the system previous to the cold weather, and remained latent, and the depressing effect of the cold had something to do in developing and manifesting the disease. It is true that many features of the disease under consideration were characteristic of diseases arising from such a cause.

The symptoms were nearly the same as those of bilious fever; it resembles it in duration, and in its general tendencies; and, while I entertain such an opinion, I know that many good practitioners of medicine, diagnosed it to be typhoid in character, erysipelas, or erysipelas with a typhoid complication, so to speak. But when we contrast the disease in question with typhoid, we find they differ widely in symptoms, duration, and final results. It is claimed that the disease, like bilious, runs into typhoid after the seventh day, and especially if it runs on to the thirteenth or twentieth, but the same gentlemen would be rather slow to believe that typhoid runs into bilious. And why not, if both diseases are produced from the same cause? The typhoid symptoms are as likely to be primary as secondary, and if bilious fever is disposed to run into typhoid, typhoid as surely runs into bilious.

Public opinion gives the physician no credit who treats a case that runs from fifteen to twenty days, unless he calls it typhoid. This is wrong. The physician who treats bilious fever, and does it successfully, is as much entitled to credit as though he treated typhoid. In typhoid fever, without entering into detail in regard to symptoms, the secretions are all increased; there is the pea-soup diarrhœa; the copious perspiration, and sudamina; the pulse is but little accelerated; the muscles become relaxed; the constitution of the blood is impaired, and the general tendencies of the system are to wasting and softening, which is evidenced after death by handling the liver, which is found to be easily torn; the muscles are softened; the bowels are ulcerated. Not so with the disease under consideration, the secretions are all locked up; the hearts actions are increased, at times so as to send the blood through the system without the proper secretion being made, therefore they are retained; the action of the skin, kidneys, liver, and bowels, are diminished, while the action of the lungs are increased, they having to throw off all the effete matter, in the shape of carbonic acid and water, in order to carry on respiration, and supply the system with oxygen. But then vicarious action does not suffice but in part, they cannot fully perform the functions of the kidneys and liver, the bile is retained, and, after a few days, unless proper remedies are used, the patient becomes jaundiced. If the constituents of the urine are retained any considerable length of time, coma is the result, and the general tendencies are to induration, inflammation, and suppuration, the disease running from seven to twenty-eight days. Unlike bilious fever, inflammation is apt to set in early about the tonsils, pleura, peritoneum, and external muscles of the neck or face; while in bilious fever, it makes its appearance, ordinarily, in the last stage of the disease, and is principally confined to the liver and spleen, and, sometimes, the meninges of the brain. But, during the epidemic of erysipelatous fever, there was no part exempt. In some of the cases which I treated at the start, it was confined to the skin, but frequently it would dip down into the cellular tissues, and, occasionally, into the muscles themselves, forming large suppurating abscesses; and the symptoms were more favorable when it located itself, than when it did not. When it made its appearance upon a part, and spent its violence there, it mitigated the constitutional symptoms, lessening the danger of breaking down the constitution of the blood, which was inevitable, if it did not locate somewhere; when it did not, the chances were against the patient. But the question may be asked, how we would know that we had erysipelatous fever, unless it was located? The answer is easy:—by the quick pulse, and wandering condition of the mind at night. I have frequently found the pulse 130 per minute, with high delirium, at night, and in the morning find the pulse not above 100 per minute, and the mind clear, and the patient flattering herself that she was, in a manner, well—for the women seemed most liable to be attacked, though no age, sex, or condition of life was exempt. I treated it in the new-born infant, to extreme old age, both male and female, but when night came around, the quick pulse and the delirium returned, prostrating the patient in a fearful manner, which is peculiar to no other disease. Sometimes patients died of nervous prostration, within 48 hours after the attack, but more usually the disease came on with chilly sensations, lassitude, pain in the head, back, and limbs, the appetite was impaired, with indisposition to exertion, this state of things continuing from 24 to 36 hours, when the patient would be seized with a severe chill, intense thirst, nausea and vomiting, with restlessness, and a great sense of muscular prostration. The tongue was usually dry and coated; skin hot; pulse strong and frequent; urine sweet and high colored; and, as the disease progressed, the system became more and more exhausted, until the disease found a location, or broke down the constitution of the blood, and resulted in death.

The next question arises, does erysipelas ever attack the internal organs? My opinion is, that it does, when prevailing epidemically, and I will here give a few cases as evidence without arguing the point:—

CASE I. Was that of a lady, about 30 years of age, who was seized with a chill, which was soon followed with high febrile excitement, with nausea and vomiting. The nausea and vomiting was very intractable, having all the appearances of gastritis, which continued for about four days, when the stomach was suddenly relieved, and there was pleuritis of the right side for about two days, when the throat became suddenly involved, the diesase passing to the parotid gland, and spreading over the entire face and part of the scalp.

CASE II. Was that of a young girl, in her seventh year, who was indisposed for a day or two, when she was attacked with shivering and severe rigors, alternating with flushes of heat. Next day there was tenderness over the umbilical region, and tympanites, which remained about four days, and had all the characteristics of peritonitis, when the patient was seized with nausea and vomiting. I examined the abdomen and found the tenderness had subsided. During the next 24 hours the nausea and vomiting had ceased, and the cervical muscles were swollen and tender, and the disease soon made its appearance upon the face and scalp.

CASE III. Was that of a woman, about 35 years of age, who was taken, apparently, with pleuritis of a very violent character. There was no cough or expectoration, but the pain was severe, with great tenderness along the side of the chest, with high febrile excitement, great thirst, and a wandering condition of the mind at night. Therefore, I decided it to be erysipelas. About the seventh day, when I had given up all hope, the inflammation passed up into the throat, and from there to the face, and the patient speedily recovered.

But such happy results were not always to be looked for. When the disease attacked the peritoneum, mucous membrane of the stomach, pleura, or meninges of the brain, it was apt to remain there until the parts were destroyed, and death was the result. The inflammation had a strong tendency to suppuration, in the majority of the cases I treated of the erratic form of the disease; abscesses would form about the angle of the jaw, and sometimes the tonsils had to be opened, being the seat of large abscesses. One patient, that died of peritonitis, I bled two days before death, but discovered nothing except that the blood was sizy and buffed, consisting of a large amount of serum and a small clot.

The disease did not seem to be contagious, but usually went through families. There were several cases that were thought to be produced by inoculation. In regard to treatment, the most successful was alteratives, tonics, stimulants, and anodynes. Local applications, in general, were nothing more than placebos. At the commencement of the disease calomel and jalap, and Dover's powder were given, to clear the intestinal tube, and were followed with tonics, after the bowels had been thoroughly evacuated. Quinine and the tincture of chloride of iron, was the tonic I preferred, administered during the day with wine or brandy for a stimulant. The chlorate of potassa was used freely, for its solution in the blood, to facilitate the absorption of oxygen into the system, which is so much demanded where there is much nervous prostration. After the disease has progressed two or three days, tartar emetic is an invaluable remedy, given in about half grain doses, just enough to produce emesis, it relaxes the system, and establishes all the secretions, better than even calomel itself, and there is no danger of ptyalism, which is much to be dreaded, and, especially, if there is a tendency of the disease to the throat; it overcomes the action of the heart, mitigating the force of the circulation, giving the kidneys time to perform their proper function; it arouses the bile cells in the liver to action, causing large quantities of bilious matter to be evacuated, especially where there is a bilious complication, which is apt to be the case either primary or secondary, but in the majority of cases it is primary—that is one of the first things that demands our attention. During the night, morphia enough to calm the restlessness and produce sleep is sufficient, more than that seeming to have a deleterious effect, rather than otherwise, locking up the secretions of the kidneys and liver after they have been established, counteracting, to some extent, the effect of the alterative part of the treatment, which is the main dependence to be relied upon for the cure and restoration of the patient to health. As an alterative, the syrup of iodide of iron was made use of, but its action seemed to be too slow, or did not seem to control the disease as well as the tincture of chloride of iron combined with the sulphate of quinine. But I do not think it safe to depend upon the tincture of iron alone in the treatment of erysipelas while prevailing epidemically, and especially where there is a bilious complication, notwithstanding it has been lauded as the best remedy, and I have no doubt but it is an efficient remedy enough in the idiopathic form of the disease, but when there is a marked periodicity about the disease, I would prefer it combined with sulphate of quinine, as above stated. The sulphate of quinine being, in my opinion, an indispensable remedy as a tonic, and anti-periodic, and about the only trustworthy antidote to the poison of the zymotic or morbific agent that originates the disease, by fermentation, or otherwise, it should be omitted during the night, when the delirium is high, but through the day it can be given with impunity. The local use of the tincture of iodine, in the erratic form of the disease, has been highly recommended by physicians both in Europe and in the United States. But, after a faithful trial, it did not come up to my expectations. However, in one or two cases it seemed to do good in arresting the progress of the disease, yet it was impossible to say whether the iodine arrested it or the constitutional treatment, for I never trusted it alone but in one case, and that was a young infant, about two weeks old, that could not take a constitutional treatment.

The disease first made its appearance in the vagina, and over the perineum, and followed the inguinal region to the abdomen, which it entirely covered. The tincture of iodine was painted over the parts, first in a diluted form, and lastly in full strength, but did not arrest the disease, and there was no constitutional disturbance at the start, apparently, of any consequence. In the majority of the cases, where the face was attacked, the disease disappeared as soon without its use as with it, depending entirely upon the constitutional treatment and the length of time after the attack before it located itself upon the face.

